# Doubly Constrained C-terminal of Roc (COR) Domain-Derived Peptides Inhibit Leucine-Rich Repeat Kinase 2 (LRRK2) Dimerization

**DOI:** 10.1021/acschemneuro.3c00259

**Published:** 2023-05-18

**Authors:** Pragya Pathak, Krista K. Alexander, Leah G. Helton, Michalis Kentros, Timothy J. LeClair, Xiaojuan Zhang, Franz Y. Ho, Timothy T. Moore, Scotty Hall, Giambattista Guaitoli, Christian Johannes Gloeckner, Arjan Kortholt, Hardy Rideout, Eileen J. Kennedy

**Affiliations:** † Department of Cell Biochemistry, University of Groningen, Nijenborgh 7, 9747AG Groningen, Netherlands; ‡ Department of Pharmaceutical and Biomedical Sciences, College of Pharmacy, University of Georgia, Athens, Georgia 30602, United States; § Center for Clinical, Experimental Surgery, and Translational Research, Biomedical Research Foundation of the Academy of Athens, 11527 Athens, Greece; ∥ YETEM-Innovative Technologies Application and Research Centre, Suleyman Demirel University, 32260 Isparta, Turkey; ⊥ DZNE German Center for Neurodegenerative Diseases, 72076 Tübingen, Germany; # Core Facility for Medical Bioanalytics, Center for Ophthalmology, Institute for Ophthalmic Research, University of Tübingen, 72076 Tübingen, Germany

**Keywords:** constrained peptides, LRRK2, Parkinson’s disease, kinase, stapled peptide, allosteric inhibition

## Abstract

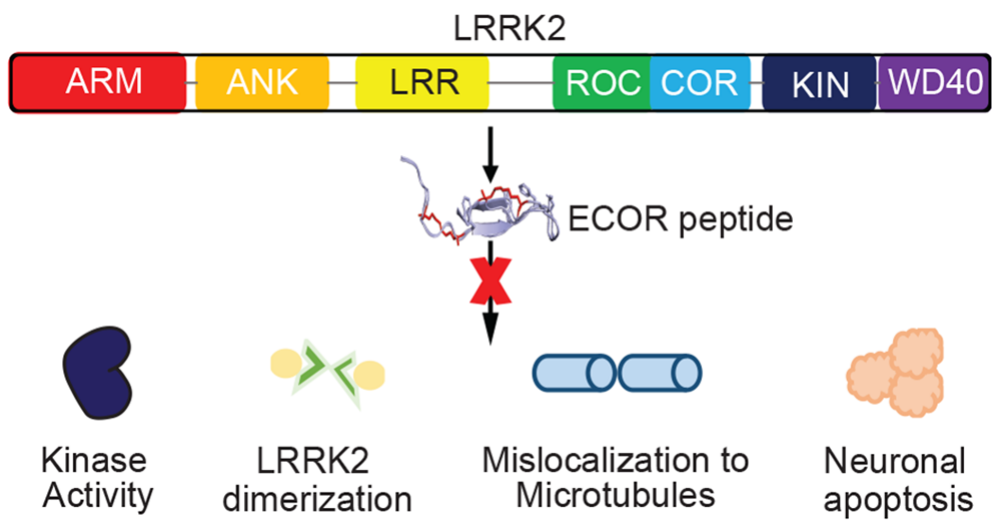

Missense mutations along the leucine-rich repeat kinase 2 (LRRK2) protein are a major contributor to Parkinson’s Disease (PD), the second most commonly occurring neurodegenerative disorder worldwide. We recently reported the development of allosteric constrained peptide inhibitors that target and downregulate LRRK2 activity through disruption of LRRK2 dimerization. In this study, we designed doubly constrained peptides with the objective of inhibiting C-terminal of Roc (COR)–COR mediated dimerization at the LRRK2 dimer interface. We show that the doubly constrained peptides are cell-permeant, bind wild-type and pathogenic LRRK2, inhibit LRRK2 dimerization and kinase activity, and inhibit LRRK2-mediated neuronal apoptosis, and in contrast to ATP-competitive LRRK2 kinase inhibitors, they do not induce the mislocalization of LRRK2 to skein-like structures in cells. This work highlights the significance of COR-mediated dimerization in LRRK2 activity while also highlighting the use of doubly constrained peptides to stabilize discrete secondary structural folds within a peptide sequence.

The leucine-rich repeat kinase 2 (LRRK2) protein is a large multidomain protein consisting of seven domains comprising 2527 amino acid residues.^1^ Each domain along the LRRK2 protein partakes in both individual and overlapping functions. From the N-terminus to the C-terminus, LRRK2 contains armadillo repeats (ARM), ankyrin repeats (ANK), leucine-rich repeats (LRR), Ras of complex (ROC) domain, C-terminal of Roc (COR) domain, kinase domain, and WD-40 domain. LRRK2 is expressed in diverse tissues including the brain, lungs, kidney, and a subset of immune cells. In cells, LRRK2 is present as both a monomer and a dimer. In its monomeric form, the protein is largely distributed throughout the cytosol, whereas in its dimeric form, the protein is localized to specific cell organelles and membranes and performs discrete functions.^2^ Missense mutations within LRRK2 are the most common cause of genetically associated Parkinson’s disease (PD).^3^ The motor symptoms of PD are caused by the loss of dopamine-producing nerve cells in the substantia nigra in the ventral midbrain.^4,5^ PD-related pathogenic mutations along LRRK2 are mostly localized to the ROC, COR, and kinase domains.^5^ Each of these mutations alters kinase and GTPase activity which has downstream cell signaling effects including altered lysosomal maintenance, cell apoptosis, disrupted mitochondrial function, and altered vesicular trafficking, all of which are pathologies of PD.^5^ Efforts to develop LRRK2 inhibitors have largely focused on small molecule ATP competitive-binding kinase inhibitors such as MLi-2 and DNL201.^6,7^ While these small molecule inhibitors are successful at downregulating LRRK2 kinase activity, there was some evidence of toxicities observed that may be limited to particular animal models.^6,8^

Protein–protein interactions (PPIs) are a major driving force for the activation of many cellular pathways and associated disease pathologies, making them an attractive target for drug discovery.^9^ It is a significant challenge to design small molecules that inhibit PPIs for multiple reasons including their small inherent size relative to the large hydrophobic surfaces that often encompass PPI interfaces.^10^ As an alternative approach, we previously developed constrained peptides derived from the Roc domain of LRRK2 to target and allosterically inhibit the dimer interface.^11^ A second peptide used in this previous study targeting the COR domain showed limited cell uptake, weak binding affinity, and limited cellular activity as compared to the Roc-targeting peptide. However, earlier studies on Roco proteins demonstrated the significance of the COR domain in protein dimerization.^12^ In addition, a recently published full-length structure of the inactive LRRK2 dimer revealed that the COR domain comprises a significant portion of the LRRK2 dimer interface and may play a key role in mediating dimerization.^13^ In this structural study, the LRRK2 COR–COR dimer interface was predominately composed of stacked β sheets that form a “7 + 1” structure with seven β strands from one COR subunit stack and one β strand from the second COR subunit through hydrophobic interactions.^13^ Further, the previous homology model of the cryo-EM structure of the C-terminal portion of LRRK2 containing the ROC–COR–kinase–WD40 domain (LRRK2^RCKW^) portion of LRRK2 also identified the COR domain as a key component for LRRK2 dimerization.^14^ Taken together, both structures highlight an important role for the COR domain in LRRK2 dimerization.

In this study, we report the design and synthesis of doubly constrained peptides that mimic the first sheets in the “7 + 1” interface of the COR–COR dimer. These doubly constrained peptides permeate cells, bind to both wild-type and PD-associated pathogenic forms of LRRK2, inhibit LRRK2 dimerization, downregulate LRRK2-mediated kinase activity, inhibit LRRK2-mediated neuronal apoptosis, and do not induce mislocalization of LRRK2 to skein-like structures in cells. This study supports the hypothesis that the COR–COR dimer interface is critical for LRRK2 dimerization and provides an alternative strategy to LRRK2-targeted PD therapy. Further, we demonstrate that the addition of a single staple was not sufficient for cell permeation and the second staple was necessary for cell-based experiments, highlighting an additive impact of a second staple for promoting cell permeation.

## Results and Discussion

### Design of Doubly Constrained Peptide Inhibitors Destabilizing COR–COR-mediated Dimerization of LRRK2

**Figure 1:**
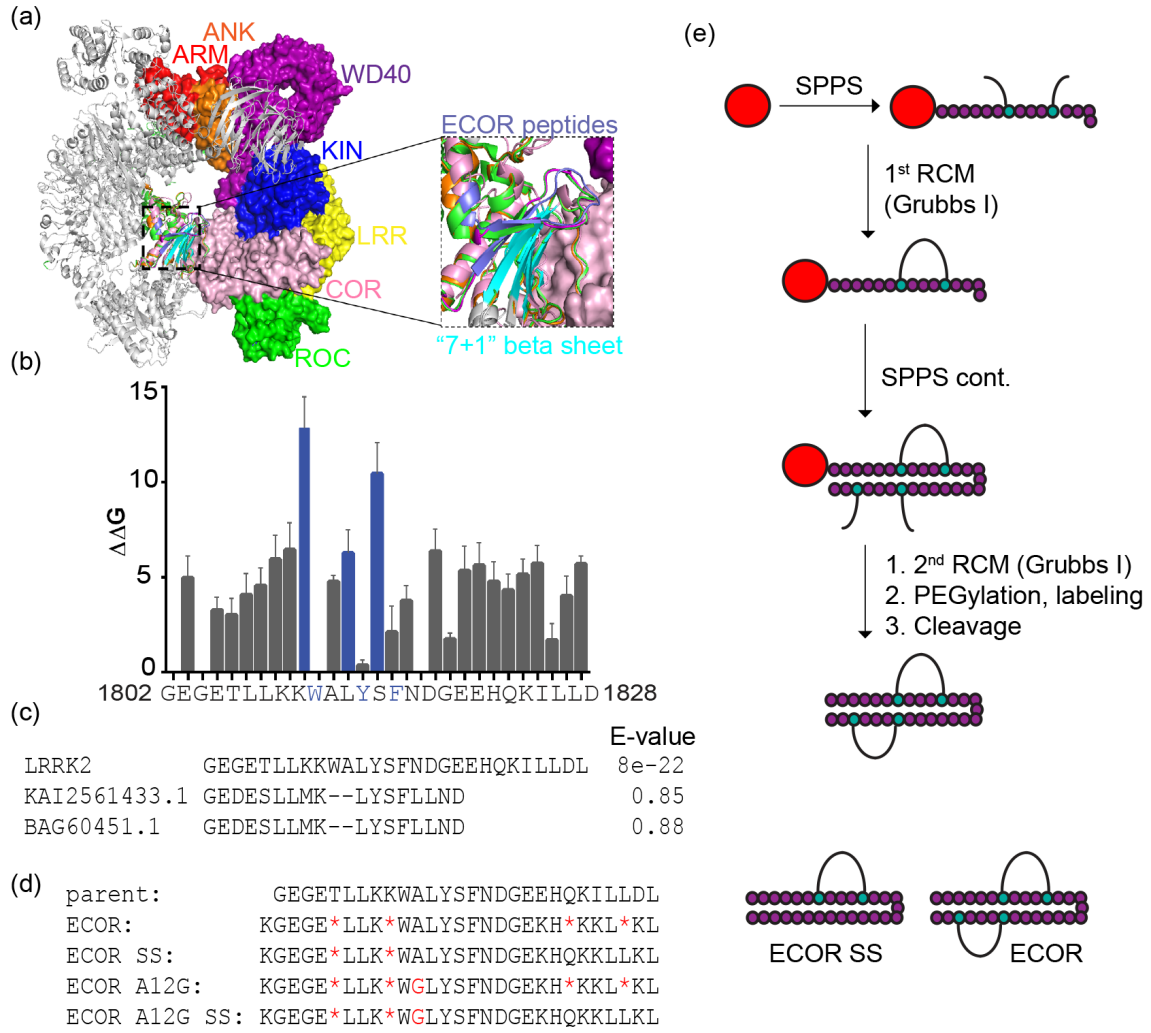
Design and Synthesis of the Elongated COR (ECOR) Peptide Library. (a) Structure of the LRRK2 dimer interface highlighting the targeted COR:COR interface (shown in pink). The “7 + 1” stacked beta sheets are shown in teal. The ECOR peptide sequence is shown in lilac. (b) Schematic representation of the *in silico* alanine scan results for the ECOR peptide sequence residues (residues 1802−1828, *n* = 10 models). Shown in navy blue is the hydrophobic triad that was identified as critical for binding. (c) Blastp results demonstrate that this sequence is unique to LRRK2 with the nearest proteins having low homology to this sequence. (d) The ECOR peptide library is shown. Red asterisks indicate the placement of the olefinic amino acid S_5_. The glycine substitution is also shown in red (A12G). (e) Schematic of synthetic strategy to generate singly and doubly constrained hydrocarbon stapled ECOR peptides through solid-phase-peptide-synthesis (SPPS).

Some differences between the recently solved structures of full-length LRRK2 and the truncated LRRK2^RCKW^ structure highlight the potentially dynamic nature of the LRRK2 dimerization interface as it transitions between monomeric and dimeric states.^13,14^ In addition, structural changes may occur as the kinase domain transitions from an inactive to an active conformer. The full-length structure of LRRK2 was used as a starting point for PPI inhibitor design where key protein interactions in both the monomeric and a dimeric state were revealed.^13^ Further, the dimer structure suggested a potentially important interaction within the terminal β sheets of the “7 + 1” COR dimer hydrophobic interface. Based on these interactions, we sought to design constrained peptides that mimic a portion of this interface as a strategy to disrupt the packing interactions at the PPI, thereby resulting in inhibition of COR–COR-mediated LRRK2 dimerization. We designed a series of peptides that mimicked the C-terminal portion of the COR domain consisting of a portion of the “7 + 1” sheet stacking interface (residues 1802–1828) [Figure 1A]. Using this sequence, we performed an *in silico* alanine scan (BudeAlaScan) to identify residues that appear to be critical for binding the targeted PPI.^15,16^ A hydrophobic triad (1811W, 1814Y, and 1816F) was identified from this scan that was predicted to serve as high energetic contributors to binding [Figure 1B]. To determine whether this sequence may be unique to LRRK2, a blastp search was performed [Figure 1C]. This sequence was found to be specific to LRRK2 where the nearest proteins had low homology to this region with E-values of 0.8–0.9. Further, while many of the “strand” residues were predicted to contribute to binding, several of the “turn” residues were found to be less critical for binding. Olefinic amino acids (pentenyl alanine, S_5_) were introduced in positions that were predicted to play minor roles in binding. In addition, since this sequence contained two noncontinuous secondary structural elements, we designed a variant sequence bearing an alanine to glycine substitution at position 1812 (ECOR A12G). The aim of this substitution was to provide more flexibility at the end of the first secondary structural element to reinforce flexibility at the “loop” region. Moreover, since the overall sequence was 28 residues in length, it was unclear whether one staple would be sufficient to promote cell permeation, so two versions were generated that are singly or doubly stapled (“SS” denotes single staple) [Figure 1D]. Peptides were synthesized via Fmoc solid phase peptide synthesis on rink amide MBHA resin. Non-natural amino acids were introduced at *i*, *i* + 4 positions. In the instance of the double-stapled variants, the sequence was synthesized up to the point of the incorporation of the first pair of olefinic amino acids and the ring-closing metathesis (RCM) reaction was performed while the last incorporated amino acid was still Fmoc-protected. The remaining sequence was then completed prior to performing an additional RCM reaction to form the second macrocycle to eliminate the possibility of cross-metathesis products being formed [Figure 1E]. For all sequences, select non-critical residues within the sequence were substituted with lysine residues and an N-terminal PEG_3_ linker to improve hydrophilicity and overall net charge. Upon cleavage from resin, peptides were purified using RP-HPLC and products were confirmed by ESI-MS [Figures S1–S7].

### Doubly Constrained Peptides Directly Bind to LRRK2

**Figure 2:**
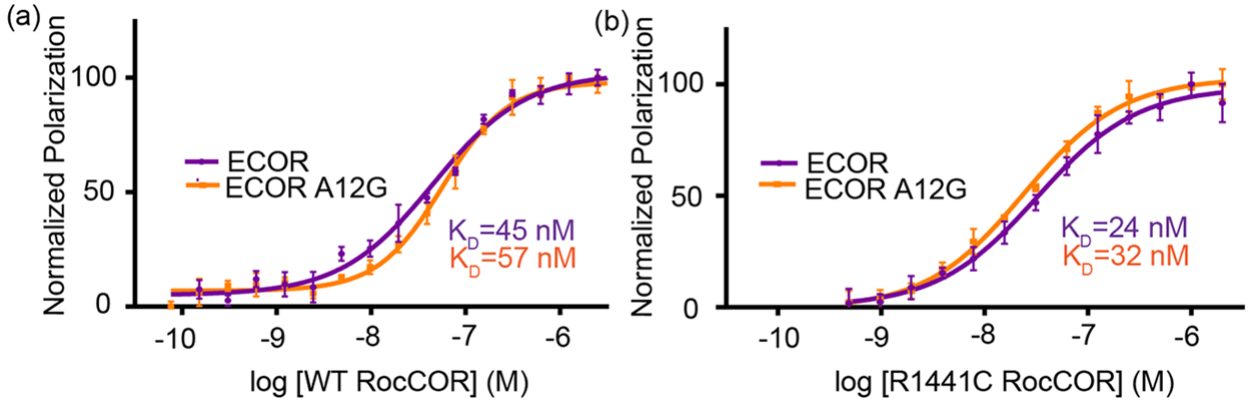
Doubly constrained peptides bind the RocCOR domain of LRRK2. (a) Fluorescence polarization (FP) assays using doubly constrained FAM-labeled peptides (ECOR and ECOR A12G) and the RocCOR domain of LRRK2 demonstrate that both peptides bind this construct with *K*_D_ values ranging from 45 to 60 nM. (b) FP assays were performed using a RocCOR construct bearing the R1441C disease-associated mutation. Peptides bound this construct with higher affinities with *K*_D_ values ranging between 25 and 35 nM. Data is representative of triplicate experiments.

We first sought to determine whether the peptides could bind to the LRRK2 RocCOR domain. We performed fluorescence polarization (FP) assays using either the wild-type RocCOR domain or a pathogenic mutant form (R1441C) in the presence of fluorescently labeled, doubly stapled versions of ECOR [Figure 2A,B]. Both ECOR and ECOR A12G exhibited binding with dissociation constants between 45 and 60 nM for wild-type RocCOR. Further, we observed that both ECOR and ECOR A12G displayed slightly stronger binding toward pathogenic LRRK2 as compared to the wild-type binding with *K*_D_ values ranging from 25 to 35 nM. Together, this demonstrates that the doubly constrained ECOR peptides can bind the RocCOR domain of LRRK2 with relatively high affinities.

### Peptides are Resistant to Proteolytic Degradation

**Figure 3:**
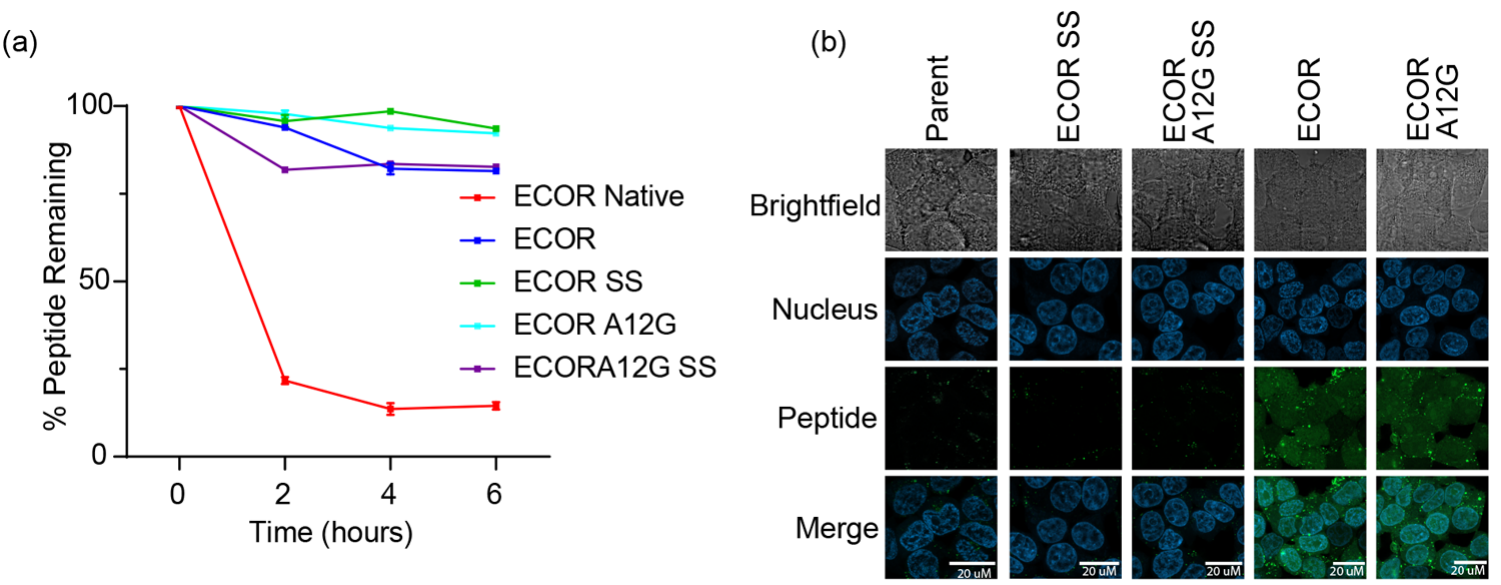
Stapled peptides are resistant to proteolytic degradation, but only doubly constrained peptides permeate cells. (a) Proteolytic stability was measured for each peptide using fresh cell lysates over a 6 h time course at 37 °C. The singly and doubly stapled peptides both demonstrate considerable proteolytic stability with over 80% integrity remaining after the 6 h time point, while the unstapled parent peptide only retained 15% of its integrity after 6 h. Plots are representative of triplicate experiments. (b) HEK293 cells were treated with 2.5 μM of each FAM-labeled peptide for 6 h at 37 °C. Doubly stapled peptides were found to permeate cells, while the singly stapled peptides and the unstapled parent control peptide did not.

Since these peptide sequences are relatively long, we sought to determine whether they may be vulnerable to proteolytic degradation. To assess stability, the peptide library was incubated with freshly prepared cell lysates to measure proteolytic degradation over time as analyzed by mass spectrometry [Figure 3A].^17^ Stability was assessed over a 6 h time course, whereby the amount of peptide remaining over time was analyzed by ESI-MS using benzoic acid as an internal control. As expected, the nonconstrained parent peptide was readily degraded with less than 20% detected by 2 h and nearly completely degraded at the 4 h time point. On the other hand, both doubly stapled peptides (ECOR and ECOR A12G) were shown to be highly stable with over 80% remaining after 6 h. In addition, the singly stapled peptides were also stable under the conditions tested with comparable levels of mild degradation as compared to the doubly stapled versions. In addition, peptide stability was also measured using mouse serum and analyzed by ESI-MS [Figure S8]. Similar results were found where the nonconstrained parent peptide was rapidly degraded within 1 h while the constrained peptides were stable over the full time course.

### ECOR and ECOR A12G Permeate Cells

LRRK2 is localized within the cell’s intracellular space, therefore we monitored whether the constrained peptides could permeate cells. HEK293 cells were incubated in the presence of peptides at 37 °C for 6 h prior to imaging by confocal microscopy [Figure 3B]. While the single stapled peptides demonstrate low levels of cellular uptake compared to the native peptide, the uptake of doubly constrained peptides is substantially higher as determined by fluorescence intensity. These findings demonstrate the significance of the second hydrocarbon staple in improving cell permeation for this peptide sequence.

### ECOR and ECOR A12G Downregulate LRRK2 Dimerization

**Figure 4:**
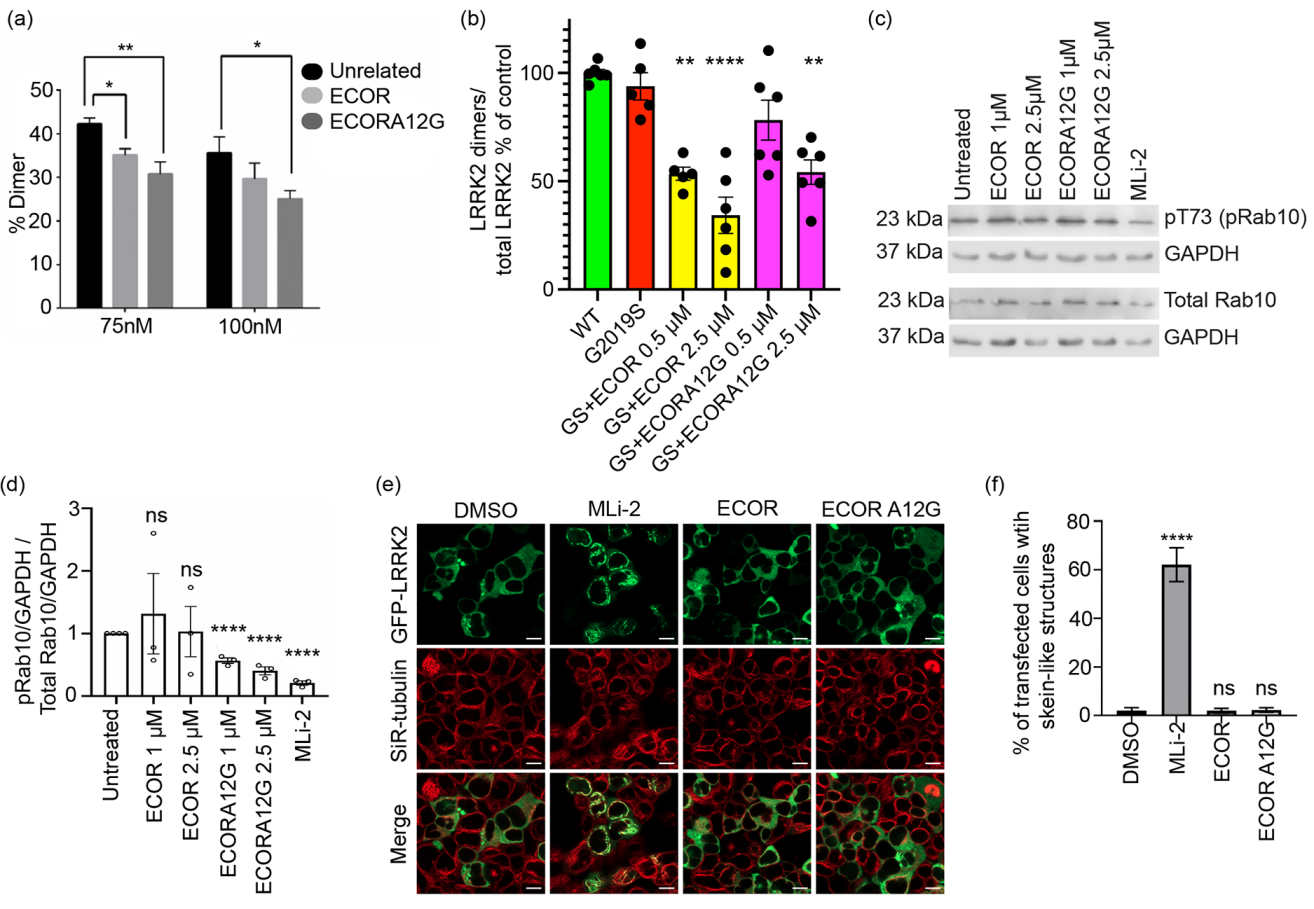
Doubly constrained peptides downregulate LRRK2 dimerization and LRRK2 kinase activity and do not cause microtubule mislocalization of LRRK2. (a) Mass photometry was applied to determine the molecular weight of LRRK2 to measure monomer and dimer species. Purified full-length LRRK2 was incubated with 7.5-fold excess of peptide for 30 min at 30 °C. The percentage of dimer for the different conditions is depicted. Full diagrams are provided in Figure S9. *p < 0.05, **p < 0.01. (b) A proximity biotinylation assay was used to measure LRRK2 dimerization in cells. Both doubly constrained peptides downregulated dimerization by approximately 50%. Experiments were performed in triplicate; ns not significant, *p < 0.05, **p < 0.005, ****p ≤ 0.0001. (c) Western blot analysis of Rab10 phosphorylation in A549 PPM1H knockout cells treated with different concentrations of doubly constrained peptides demonstrates downregulation of LRRK2 kinase activity as compared to the untreated control after incubation for 6 h. (d) Quantification of mean intensity of phospho-Rab10 signal normalized to GAPDH over total Rab10 signal normalized to GAPDH, with standard error of mean (SEM) for at least three independent experiments. An unpaired 2-tailed *t* test was performed; ****p ≤ 0.0001, ns not significant (*p* > 0.05). (e) HEK293 cells were transfected with GFP–LRRK2 for 24 h and subsequently treated with DMSO, 1 μM MLi-2, or 2.5 μM ECOR or ECOR A12G peptide for 6 h at 37 °C. The cells were treated with media containing SiR-tubulin for tubulin staining and verapamil (a broad-spectrum efflux pump inhibitor). The cells treated with MLi-2 show skein-like filamentous structures that overlap with tubulin filaments stained by SiR-tubulin. Scale bar 10 μm. (f) A minimum of 500 transfected cells were quantified for skein-like structures for each condition as shown in panel c. The graph represents the average percentage of cells, and standard errors of the mean (SEM) for three independent experiments with at least two biological replicates are shown with *p* values: One-way ANOVA and Dunnett’s multiple comparisons test (DMSO as a control), **** *p* ≤ 0.0001, ns not significant (*p* > 0.05).

Since the peptides were derived from the COR–COR interface of the LRRK2 dimer, we sought to assess whether these peptides could effectively inhibit LRRK2 dimer formation using mass photometry. Purified full-length LRRK2 was incubated for 30 min with 7.5-fold excess of each doubly constrained peptide [Figures 4A and S9]. The molecular weight of dimeric LRRK2 was used as a method of assessment for LRRK2 dimeric presence. Both peptides inhibited the formation of LRRK2 dimer at both 75 nM and 100 nM, with the strongest dimer inhibition seen for ECOR A12G. These findings demonstrate that these COR-interface derived doubly constrained peptides inhibit LRRK2 dimerization.

In addition, we measured whether the peptides could inhibit LRRK2 dimerization within the cellular environment. For this, we used the previously published *in situ* LRRK2 proximity biotinylation approach where LRRK2 constructs bearing either BirA or an acceptor peptide are cotransfected, and the level of biotinylated LRRK2 is measured.^18^ Both ECOR and ECOR A12G were found to downregulate dimerization of a PD-related pathogenic form of LRRK2 (G2019S) that exists primarily in the dimeric form [Figure 4B]. Doubly constrained peptides were tested over a concentration range of 0.5–2.5 μM and were found to downregulate intracellular LRRK2 G2019S dimerization by approximately 50–60%, demonstrating that these COR interface-derived peptides can indeed prevent LRRK2 dimerization.

### ECOR and ECOR A12G Downregulate LRRK2 Kinase Activity but Do Not Induce Microtubule Mislocalization of LRRK2

Since LRRK2 kinase activity is linked to LRRK2 dimerization,^18−20^ we aimed to determine whether the COR-derived peptides downregulate this kinase activity as a function of disrupted dimerization. A549 PPM1H knockout cells were treated with 1 μM and 2.5 μM ECOR and ECOR A12G for 6 h. Lysates were analyzed by Western blotting to probe for phosphorylation of the LRRK2 substrate Rab10 [Figure 4C,D]. At both concentrations, ECOR A12G was found to downregulate Rab10 phosphorylation by nearly 40% while ECOR was not found to have measurable effects on substrate phosphorylation. When compared to the small molecule ATP-competitive kinase inhibitor MLi-2, which nearly completely inhibited Rab10 phosphorylation, neither of the doubly constrained peptides was as potent; however, using an allosteric approach, ECOR A12G can downshift the kinase activity of LRRK2.

Classical ATP-competitive LRRK2 kinase inhibitors induce cellular recruitment of LRRK2 to skein-like structures on microtubules and block kinesin and dynein-1-mediated transport *in vitro*, which might partly induce the side-effects reported for these compounds [Figure 4E,F].^14,21^ To test whether peptides would have a similar effect, GFP-tagged LRRK2 was overexpressed in HEK293 cells for 24 h. The cells were then incubated at 37 °C for 6 h with either DMSO, MLi-2, ECOR, or ECOR A12G prior to live imaging. The cells were treated with a tubulin stain to measure tubulin colocalization. Although it was previously reported that cells overexpressing LRRK2 show some aggregation,^21^ in the presence of the ECOR and ECOR A12G peptides, no significant localization of LRRK2 to microtubules as filamentous skein-like structures could be observed. On the other hand, MLi-2-treated cells demonstrated considerable colocalization with tubulin. Thus, it appears the peptides do not induce microtubule mislocalization of LRRK2 in cells.

### ECOR and ECOR A12G Downregulate Mitochondrial Oxidative Stress

**Figure 5:**
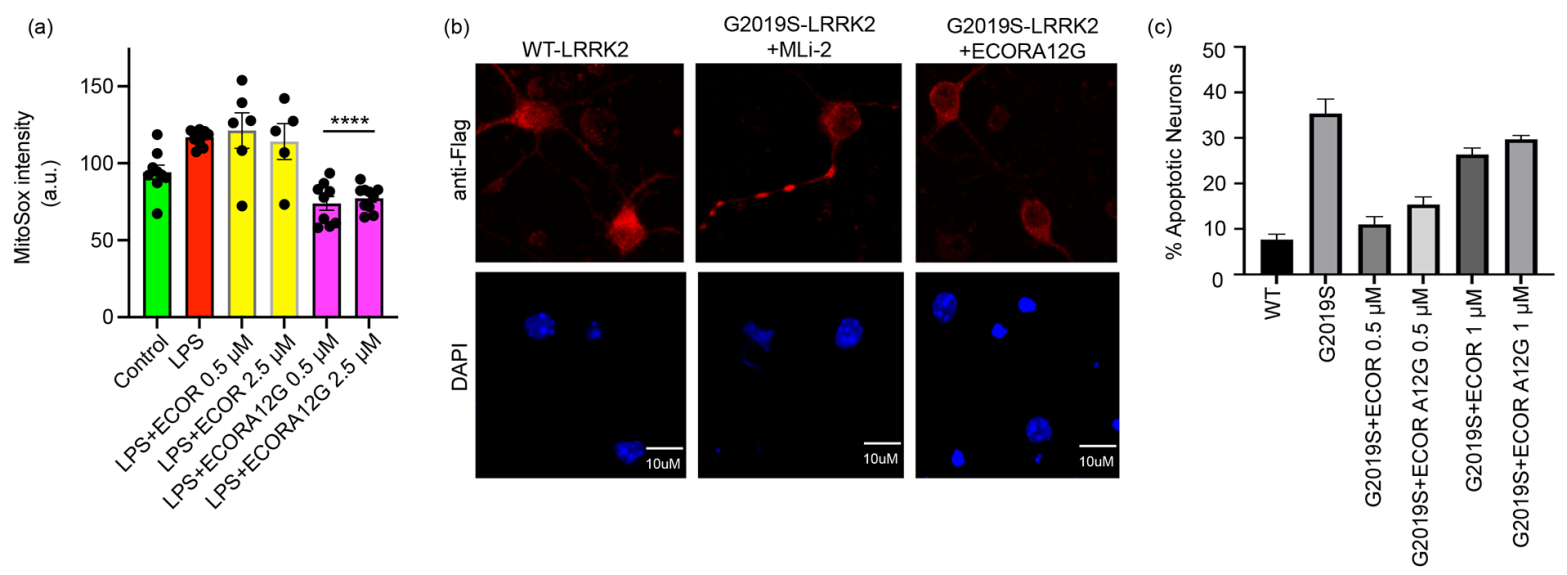
Doubly constrained peptides downregulate LRRK2-mediated neuronal apoptosis. (a) Assessment of mitochondrial oxidative stress. RAW264.7 cells were treated with lipopolysaccharide (LPS) and peptides for 3 h. Both concentrations tested for ECOR A12G reduced the production of oxidative stress by greater than 25% as compared to the untreated control. (b) Representative images of primary cortical neurons expressing wild-type LRRK2 and LRRK2 G2019S that are treated with 0.5 μM ECOR A12G and MLi-2. Nuclear condensation and fragmentation are reduced after peptide treatment. (c) Quantification of apoptotic primary neurons expressing WT LRRK2 and G2019S LRRK2 with or without peptide treatments. Primary neurons were transiently transfected with Flag-LRRK2 (either WT or G2019S), followed by treatment with 0.5–1 μM ECOR or ECOR A12G for 48 h. Both doubly stapled peptides greatly reduced neuronal apoptosis with 0.5 μM peptide treatments. Neurons from three separate biological replicates were counted in a blinded manner.

Upregulated LRRK2 activity is linked to a variety of phenotypes including increased oxidative stress. To determine the effects of the doubly constrained peptides on mitochondrial oxidative stress, both peptides were incubated with lipopolysaccharide (LPS)-stimulated RAW264.7 cells for 3 h. Two separate concentrations of 0.5 μM and 2.5 μM of ECOR and ECOR A12G were used [Figure 5A]. Incubation of both concentrations of ECOR A12G resulted in a 25% reduction in mitochondrial oxidative stress compared to the other conditions. Together, these findings suggest that ECOR A12G can reduce mitochondrial oxidative stress.

### ECOR and ECOR A12G Inhibit LRRK2-Induced Neuronal Apoptosis

We next sought to determine if our lead compounds could downregulate a downstream pathology related to pathogenic LRRK2, namely, neuronal apoptosis.^22−24^ To test whether these COR-derived peptides could provide a neuroprotective effect, we measured whether they could inhibit neuronal apoptosis in isolated primary cortical neurons [Figure 5B]. Cortical neurons expressing LRRK2 G2019S demonstrated hallmarks of apoptosis including nuclear condensation and fragmentation as well as activation of caspase-3 (not shown). Both doubly constrained peptides (ECOR and ECOR A12G) were found to reduce cortical neuronal apoptosis by over 50% in the low nanomolar ranges [Figure 5C]. This reduction in neuronal cell death was most pronounced using 500 nM concentrations of either peptide. This effect may be due to insolubility or aggregation of the peptides at higher concentrations. However, it is apparent that the doubly constrained peptides can reverse the neuronal apoptotic effects that are otherwise driven by pathogenic LRRK2.

## Conclusion

Recently, we reported on the development of constrained peptides, each containing a single hydrocarbon staple, targeting the proposed dimer interface in the RocCOR domain of LRRK2 which were able to disrupt dimerization and kinase activity of LRRK2.^11^ We demonstrated that constrained peptides derived from the Roc domain of LRRK2, termed LRIP, could effectively target and allosterically inhibit dimerization, thereby downregulating kinase activity. We additionally developed a second constrained peptide that was designed to target the COR domain, termed LCIP, but this peptide had a weak binding affinity for the RocCOR domain, showed limited cell uptake, and had inferior cellular activity as compared to the Roc-targeting peptide.^11^ This finding was unexpected since recent structural studies demonstrate that the COR domain is a critical component of the LRRK2 dimer interface and thus is likely crucial for dimerization.^13^

The goal of the current study was to increase the potency of newly developed COR-derived peptides that had improved cellular uptake and efficacy while utilizing the recently characterized cryo-EM structures of full-length LRRK2 as a guide for peptide design.^13,14^ These structural studies confirmed and clarified the importance of the COR domain in facilitating LRRK2 dimerization. The peptides in the current study overlap with the originally designed COR-targeting peptides.^11^ While initial characterization studies of the newly designed library yielded no discernible differences in proteolytic stability or binding affinities toward the RocCOR domain, the cell permeation studies revealed unexpected results. Multiple studies have demonstrated that hydrocarbon stapling of a peptide sequence can increase cell permeability, and thus we expected either singly or doubly constrained peptides to permeate cells.^25−27^ The fact that two hydrocarbon staples were required for this particular sequence demonstrates a limitation of hydrocarbon staples on cell permeation, at least in this particular instance. The ability of only the doubly stapled peptides to permeate cells could be due to a multitude of factors including the flexibility, conformation, and overall molecular weight of these peptides.

There are several key advantages of using constrained peptides to disrupt PPIs. First, since they are designed to occupy a relatively flat and large binding surface that is mediated by side chain specificity, there is considerable opportunity to yield highly selective targeting agents with reduced nonspecific, off-target effects. Second, the addition of synthetic conformational constraints on the peptide sequence allows the peptide sequence to maintain a secondary structure that may mimic the preordered binding state and may thereby reduce or eliminate an energetic penalty from undergoing a disorder-to-order transition upon binding. While the potential therapeutic use of constrained peptides represents an attractive alternative to ATP-competitive LRRK2 kinase inhibitors, there are several obstacles still to overcome including the propensity to form aggregates, challenges of generating an orally bioavailable peptide-based therapeutic and their delivery into the brain which is complicated by their inability to cross the BBB. However, peptide-based inhibitors are also valuable tools for target validation and may therefore uncover new strategies for inhibitor development.

LRRK2 kinase activity is tightly linked to its dimerization^18,19,28^ and inhibition of dimerization may serve as an effective strategy to downregulate mutant LRRK2-induced neurodegeneration in cellular and *in vivo* models. While the consequences are not yet clear, there are indications of unexpected, but on-target, effects of small molecule LRRK2 kinase inhibitors such as ATP-competitive inhibitors. For example, multiple structurally distinct LRRK2 inhibitors lead to a redistribution of LRRK2 in the cell to microtubule (MT)-associated cytoplasmic filaments^21^ and can elicit mild pathology in pulmonary lamellar cells in rodents and non-human primates.^23^ Our recent description of peptide-based, Roc-derived LRRK2 dimer disrupters showed that this redistribution of LRRK2 to cytoplasmic filaments is not universally observed following inhibition of kinase activity.^11^ Similarly, in the current study, we did not observe the relocalization of LRRK2 to cytoplasmic microtubule-bound filaments. Interestingly, however, our Roc-derived peptide data reveals that loss of phosphorylated Ser935 levels, which correlates with pharmacological kinase inhibition-induced filament formation, is not sufficient to induce this redistribution of LRRK2. Genetic inhibition of LRRK2 kinase activity, such as the K1906M/R mutation, also does not lead to redistribution of LRRK2 into microtubule-associated filaments and pS935-LRRK2 loss,^21^ further highlighting the complexity of this relationship.

Together, these results indicate for the first time that COR-derived doubly constrained peptides inhibit LRRK2 dimerization, downregulate its kinase activity, and reduce mitochondrial oxidative stress and cortical neuronal apoptosis, even in the presence of a pathogenic form of LRRK2. Further, it was found that adding a second hydrocarbon staple to this sequence greatly improved cell uptake and overall LRRK2 inhibition. This work demonstrates that doubly constrained peptides targeting LRRK2 PPIs provide an alternative approach for LRRK2-targeted therapeutics by acting as an allosteric inhibitor of LRRK2 activity via disruption of LRRK2 dimerization. This work thus also highlights the significance of the COR domain for regulating LRRK2 dimerization and kinase activity and may also demonstrate new strategic approaches for downregulating aberrant kinase activity in disease states.

## Methods

### Peptide Synthesis

All solvents used in the peptide syntheses were HPLC grade. *N*-α-Fmoc protected amino acids and rink amide MBHA resin were purchased from Novabiochem. Fmoc-11-amino-3,6,9-trioxaundecanoic acid (PEG_3_) was purchased from ChemPep. Olefinic amino acid, S_5_ ((*S*)-*N*-Fmoc-2-(4-pentenyl)alanine), labeling reagent 5,6-carboxyfluorescein (FAM), and Grubbs first generation catalyst were all purchased from Sigma-Aldrich. Labeling reagent d-biotin was purchased from GoldBio. All other synthesis reagents and organic solvents were purchased from Fisher Scientific unless stated otherwise.

Peptides were synthesized on Rink amide MBHA resin using standard *N*-α-Fmoc amino acids and following the standard Fmoc solid phase peptide synthesis. First, MBHA resin was calibrated in *N*-methylpyrrolidinone (NMP) with agitation for 10 min. Fmoc-group deprotection was carried out using 25% (v/v) piperidine in 75% (v/v) NMP solution for 25 min with agitation. After each deprotection, peptides were washed three times for 30 s in NMP prior to amino acid coupling. During each coupling reaction, 10 equiv of standard amino acid, 9.9 equiv of 2-(6-chloro-1*H*-benzotriazole-1-yl)-1,1,3,3-tetramethylaminium hexafluorophosphate (HCTU) in NMP, and 20 equiv of *N*,*N*-diisopropyl ethylamine (DIEA) were added to the resin with agitation for 45 min followed by three 30 s washes with NMP. To incorporate the olefinic amino acid, 4 equiv of S_5_ ((*S*)-*N*-Fmoc-2-(4-pentenyl)alanine), 3.9 equiv of HCTU, and 20 equiv of DIEA were added to the resin with agitation for 45 min. Following the addition of all amino acids, two separate cycles of ring closing metathesis (RCM) were performed using 0.4 equiv of first-generation Grubbs catalyst in 1,2-dichloroethane (DCE) for 1 h each. Modifications were made to the N-terminus of each sequence to improve solubility and label peptides based on experimental need. First, Fmoc-11-amino-3,6,9-trioxaundecanoic acid (PEG_3_) was added to each peptide using 4 equiv under standard coupling conditions with agitation. For fluorescently labeled peptides, 2 equiv of 5,6-carboxyfluorescein in *N*,*N*-dimethylformamide (DMF), 1.8 equiv of HCTU, and 4.6 equiv of DIEA were added to the (5/6FAM)-labeled versions of the sequences overnight with agitation. For biotin-labeled peptides, 10 equiv of d-biotin, 9.9 equiv of HCTU, and 20 equiv of DIEA were added to the resin with a 1:1 mixture of dimethyl sulfoxide (DMSO) and DMF overnight with agitation. Following labeling, all peptides were separately cleaved from the resin using a solution of 95% (v/v) trifluoroacetic acid (TFA), 2.5% (v/v) triisopropylsilane (TIS), and 2.5% (v/v) distilled water. This solution was incubated for 5 h at room temperature under constant rotation. Peptide products were precipitated in methyl-*tert*-butyl ether (MTBE) and air-dried overnight prior to purification and characterization.

Sequences of each peptide used in this study are as follows (asterisks (*) represent S_5_ residues:

FAM ECOR parent: (5/6FAM)-PEG_3_-GEGETLLKKWALYSFNDGEEHQKILLDL
FAM ECOR: (5/6FAM)-PEG_3_-KGEGE*LLK*WALYSFNDGEKH*KKL*KL
FAM ECOR SS: (5/6FAM)-PEG_3_-KGEGE*LLK*WALYSFNDGEKHQKKLLKL
FAM ECOR A12G: (5/6FAM)-PEG_3_-KGEGE*LLK*WGLYSFNDGEKH*KKL*KL
FAM ECOR A12G SS: (5/6FAM)-PEG_3_-KGEGE*LLK*WGLYSFNDGEKHQKKLLKL
BIO ECOR: (d-Biotin)-PEG_3_-KGEGE*LLK*WALYSFNDGEKH*KKL*KL
BIO ECOR A12G: (d-Biotin)-PEG_3_-KGEGE*LLK*WGLYSFNDGEKH*KKL*KL

Molecular weight of the purified peptides used in this study are as follows:

FAM ECOR parent: 3948.8 (expected mass = 3949.5)
FAM ECOR: 3978.9 (expected mass = 3979.6)
FAM ECOR SS: 4099.2 (expected mass = 4099.7)
FAM ECOR A12G: 3965.4 (expected mass = 3965.6)
FAM ECOR A12G SS: 4085.2 (expected mass = 4085.7)
BIO ECOR: 3847.1 (expected mass = 3847.63)
BIO ECOR A12G: 3833.0 (expected mass = 3833.6)

### Peptide Characterization

Following cleavage and drying, peptides were redissolved in 1 mL of methanol and filtered using a 45 μm syringe filter. Peptides were then separated using an Agilent 1200 reversed phase-high performance liquid chromatograph (RP-HPLC) with a Zorbax SB-C18 column. The RP-HPLC mobile phase linear gradient contained 0.1% TFA in 10–100% water/acetonitrile with a flow rate of 0.5 mL/min. Molecular weights were used to characterize each peptide on an Agilent 6120 single quadrupole ESI-mass spectrometer. Following confirmation of the peptide presence with ESI-MS, peptides were purified over a semipreparatory column at a flow rate of 4 mL/min. Final peptide characterizations were performed by ESI-MS. [Figures S1–S7].

All peptides were quantified using a Synergy 2 Microplate Reader (Bio-Tek). A 495 nm absorbance was used for the FAM labeled peptides with an extinction coefficient of 69 000 M^–1^ cm^–1^. A small volume of each peptide was redissolved in Tris buffer (pH 8, 10 mM) to quantify the FAM-labeled peptide concentration. To quantify the biotin-labeled peptides, an absorbance of 500 nm was utilized. A small volume of each peptide was redissolved in a 2-hydroxyazobenzen-4′-carboxylic acid–avidin cocktail (HABA-avidin) to perform these quantifications. Following quantification, peptides were dried and redissolved in an appropriate volume of DMSO.

### Fluorescence Polarization (FP) Assay

Fluorescence polarization assays were conducted on the lead doubly constrained compounds (ECOR and ECOR A12G) with two separate LRRK2 protein constructs: MBP-tagged RocCOR LRRK2 and MBP-tagged R1441C RocCOR LRRK2, both in the presence of 2 mM GTP and 10 mM MgCl_2_. Protein/peptide interactions were measured in a FP buffer (20 mM MOPS, pH 7, 150 mM NaCl, and 0.005% CHAPS) at room temperature. FAM-labeled peptides were plated in 384-well microtiter plates at a final concentration of 10 nM. Proteins were added over concentrations ranging from 5 μM to 1 nM. Each protein/peptide interaction experiment was carried out at 10 different protein concentrations in triplicate. Each protein/peptide mixture was incubated at room temperature for 2 h and the final reading was performed at the end of this 2 h period.

### Cell Culture

HEK293 cells (CRL-1573), RAW264.7 macrophages (SC-6003), and HEK293T cells (CRL-3216) were purchased from ATCC. A549 PPM1H knockout cells were a kind gift of Dr. D. R. Alessi.^29^ Dulbecco’s Modified Eagle’s medium (DMEM) was purchased from Gibco (11960044). Fetal Bovine Serum (FBS) was purchased from HyClone (SH30910.03), trypsin (25-043-Cl) was purchased from Corning, and penicillin–streptomycin was purchased from Gibco (10378016). Cells were cultured and maintained in media supplemented with 10% FBS and 1% penicillin–streptomycin at 37 °C with 5% CO_2_. Cells were passaged using 0.25% trypsin solution containing 2.21 mM EDTA, 1×, with respective neutralizing media at least twice prior to each cell assay. All experiments were carried out in triplicate at different passage numbers.

### Proteolytic Stability Using Cell Lysates

Fully confluent HEK293 cells were lysed using ice-cold nondenaturing lysis buffer (20 mM Tris HCL, pH 8.0, 137 mM NaCl, 1% Triton X-100, 2 mM EDTA) and incubated for 30 min at 4 °C. Cells were centrifuged for 20 min at 12 000 rpm, and the supernatant was collected and kept on ice. A proteolytic solution containing 0.2 mM peptide, 50% fresh cell lysate, 0.4% benzyl alcohol, and 15% DMSO in PBS was used to analyze each peptide. Experiments were conducted at 37 °C with agitation for 0, 2, 4, and 6 h. Aliquots were collected and quenched at each indicated time point using an equal volume of 0.1% trifluoroacetic acid (TFA) in acetonitrile. This solution was then centrifuged at 14 000 rpm for 5 min, and the supernatant was used for analysis. Peptide degradation was analyzed on LC-MS using a Zorbax Eclipse XDB-C18 column as a ratio of peptide to control relative to peptide at *t* = 0 at 280 nm. A flow rate of 1.0 mL/min at 45 °C was used with a 0–100% water/acetonitrile gradient containing 0.1% TFA. Each individual peptide was analyzed in triplicate for each time point.

### Proteolytic Stability Using Mouse Serum

0.2 mM peptide was incubated in a proteolytic cocktail containing 50% mouse serum, 0.4% benzoic acid, and 15% DMSO in PBS at 37 °C with agitation. At each indicated time point, aliquots of the solution were drawn, and serum was precipitated using 0.1% trifluoroacetic acid (TFA) in acetonitrile. Each precipitate was collected and centrifuged at 14 000 rpm for 5 min, and the supernatants were retained for analysis. Proteolytic degradation was monitored by LC-MS as a ratio of peptide control relative to peptide at *t* = 0 at 280 nm using a Zorbax Eclipse XDB-C18 column. A 0–100% water/acetonitrile gradient containing 0.1% TFA was used at a flow rate of 1.0 mL/min at 45 °C. This process was repeated in triplicate for each time point for each peptide.

### Peptide Uptake Assay

40 000 HEK293 cells were plated into 8-well Ibidi slide with poly(l-lysine) coating and polymer coverslip (80824). The cells were incubated in complete DMEM (10% FBS, 1% penicillin–streptomycin) overnight and subsequently treated with 2.5 μM FAM-labeled peptide at 37 °C for 6 h. Following peptide incubation, cells were fixed using 4% paraformaldehyde solution (PFA) for 20 min. 1× PBS containing DAPI was added to the cells after aspirating the 4% PFA. Cells were then stored at 4 °C overnight in the dark prior to imaging. Uptake of the FAM-labeled peptides was visualized using 63× oil-immersion objective of a Zeiss LSM800 confocal laser scanning microscope.

### Mass Photometry (MP) Assay

LRRK2 was purified as previously described.^30^ LRRK2 was diluted to a concentration of 150 nM or 200 nM and incubated with constrained peptide in a 1:7.5 ratio, for 30 min at 30 °C. Mass photometry was performed on a Refeyn Two^MP^ instrument.^30^ The instrument was first focused with 10 μL of buffer (50 mM HEPES, 150 mM NaCl, 5 mM MgCl_2_, 1 mM DTT, 0.1 mM GDP, and 1% glycerol) after which 10 μL of the incubated LRRK2 solution was added. The samples were recorded for 1 min. Three measurements were processed for each condition, and the data were analyzed by GraphPad Prism.

### Proximity Biotinylation of Dimeric LRRK2 Assay

LRRK2 dimerization was assessed using either wild-type LRRK2 homodimers or LRRK2 G2019S homodimers. Experiments were carried out using various concentrations (0.5, μM, 1 μM, or 2.5 μM) of FAM-labeled ECOR or ECOR A12G. The LRRK2 dimers used were purified as previously described.^18^ To generate these dimers, two cDNAs encoding LRRK2 fusions with biotin ligase (BirA; N-term, Flag-tagged) or an acceptor peptide (AP, N-term; c-Myc tagged) were created. These constructs were expressed in HEK293T cells grown in biotin-depleted medium (OptiMEM+2% FBS). FAM-labeled ECOR12A and ECOR12G were diluted in serum-free medium and added 24 h after transfection. 48 h following the initial treatment (after 72 h of total expression), cells were washed in PBS, given a brief biotin pulse (50 μM, 5 min, 37 °C), washed three more times in PBS, and centrifuged. The cell pellet was snap frozen in a dry ice/methanol bath and stored at −80 °C until analysis. Cells were lysed, and extracts were diluted in a TBST/BSA solution (10 mM Tris HCl, pH 7.6; 100 mM NaCl; 0.1% Triton X-100; 1% BSA) with 0.5 μg of protein loaded in parallel ELISA plates coated with streptavidin (SA; to capture biotinylated LRRK2 dimers) and anti-LRRK2 (to quantify LRRK2 expression). Protein-bound SA coated plates were incubated with HRP-conjugated anti-Flag antibodies for 1 h at room temperature to quantify dimeric LRRK2. Total LRRK2 overexpression was quantified using HRP-conjugated LRRK2 antibody (clone N241) on the parallel anti-LRRK2 coated plates (precoated with anti-LRRK2, clone c41-2) and was used to normalize dimeric LRRK2 content. Proximity biotinylation experiments were performed in biological triplicates with 3–4 technical replicates for each condition in the ELISA.

### Rab10 Immunoblotting

A549 PPM1H knockout cells were treated with 1 μM and 2.5 μM of doubly constrained peptide for 6 h. Cells treated with 1 μM MLi-2 were used as a positive control. Cell lysates were separated by SDS-PAGE and transferred to PVDF membranes by wet transfer. pRab10 T73 and total Rab10 signals were blotted on different membranes with GAPDH as a loading control on the same membrane to save the membrane from the stripping step. The membranes were blocked in nonfat milk and probed with Rab10 (phospho-T73) (Abcam; ab241060, Lot; GR327 4620-4), total Rab10 (Cell Signaling Technology; #4262S), and total GAPDH (14C10) (Cell Signaling Technology; #2118S) antibodies overnight at 4 °C, followed by incubation with HRP anti-rabbit secondary antibody, and developed using ECL. The blots were quantified using Image Studio Lite version 5.2 by selecting the bands to obtain the intensity for each band. The phospho-Rab10, total Rab10, and GAPDH signal for every sample were normalized to its untreated control for the same day. The phospho-Rab10 and total Rab10 signal was further normalized to the GAPDH signal (loading control) on the same blot. The graph was plotted with GAPDH normalized phospho-Rab10 signal over total Rab10 signal for every sample (*normalized to untreated sample).
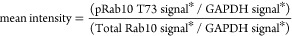


### LRRK2 Microtubule Localization Assay in Live Cells

HEK293 cells were transfected with GFP–LRRK2 for 24 h and subsequently treated with DMSO, 1 μM MLi-2, and 2.5 μM ECOR or ECOR A12G peptide for 6 h at 37 °C. The transfected cells were scored single-blinded for skein-like filamentous structures per condition. For tubulin staining, after a 6 h incubation with DMSO, MLi-2, or biotin-labeled peptides, the media was replaced with media containing 1 μM SiR-tubulin (Spirochrome SiR-tubulin Kit (SC002)) and 10 μM verapamil (a broad spectrum efflux pump inhibitor) for 1 h at 37 °C. Live cells were imaged using the 63× oil-immersion objective of a Zeiss LSM800 confocal laser scanning microscope. The Zeiss microscope software ZEN was used to generate channel overlays.

### Mitochondrial Oxidative Stress

To determine if disruption of LRRK2 dimerization reduced oxidative stress, we employed the murine macrophage cell line RAW264.7 treated with lipopolysaccharide (LPS). Cells were plated in a 12-well plate and, the following day, incubated for 3 h with LPS (500 ng/mL). At the end of the treatment period, the cells were washed and switched to phenol red-free HBSS containing the mitochondrial superoxide probe MitoSox (ThermoScientific) and incubated for 30 min at 37 °C. The plates were removed from the incubator, and live cell images were acquired for subsequent analysis using the fluorescence intensity plugin in ImageJ. At least 5 low-magnification (20×) images from each of two duplicate wells were obtained, with a minimum of 10 cells measured from each image field. The background signal from each well was subtracted from the MitoSox signal. In cells treated with FAM-ECOR peptides, cells were initially selected based on positive fluorescent FAM signal indicating uptake of the peptides; then the corresponding cells in the parallel MitoSox image were measured.

### Neuronal Apoptosis

Quantification of apoptotic neuronal profiles was performed using the approach previously described.^30^ Cortices were removed from embryonic day 16 (E16) pregnant C57BL mice and cut into small pieces for enzymatic digestion (trypsin 0.05% and 100 μg/mL DNase) and mechanical dissociation. Cells were collected, centrifuged, and grown at 150 000/cm^2^ cell density in BrainPhys neuronal culture medium (containing SM1 Neuronal Supplement, l-glutamine (0.5 mM), and penicillin/streptavidin). Neurons were then transfected using Lipofectamine 2000 with Flag-tagged WT LRRK2 or G2019S LRRK2. The following day, neurons were treated with 0.5 μM and 1 μM of both ECOR12A and ECOR12G for 48 h. Cells were washed in PBS and fixed in 3.7% paraformaldehyde for 20 min at 4 °C prior to antibody treatments. Images were analyzed and quantifications were performed as previously described.^30^ For the determination of apoptotic neuronal death, fixed and stained neurons were visualized under a 40× magnification objective (dry). On each coverslip, a minimum of 100 Flag-positive neurons were identified and the percentage of those with apoptotic profiles was determined. Apoptotic nuclei were defined as neurons having condensed chromatin, fragmented into at least 2 or more “apoptotic bodies”.

### Statistical Analysis

GraphPad Prism was utilized to perform statistical analysis. Additionally, FIJI/ImageJ, ZEN software, and Image Studio (Li-COR) were used to quantify cell uptake images, microtubule localization images and immunoblotting images, respectively. One-way ANOVA and Dunnett’s multiple comparisons test were used for the analysis of Western blots. For the analysis of the neuronal apoptosis and *in vitro* dimerization assays, one-way ANOVA with Tukey post hoc tests were performed. All experiments were performed in triplicate unless stated otherwise. Graphical data are presented as mean ± SEM.

## Supporting Information Available

Figures S1–S7: ESI-MS spectra for peptides used in this study. Figure S8: Proteolytic stability of peptides in mouse serum. Figure S9: Mass photometry diagrams for dimerization assay (PDF)

## Abbreviations

LRRK2: leucine-rich repeat kinase 2
PPI: protein–protein interface
BBB: blood–brain barrier
