# Supplementary material for: Doubly Constrained C-terminal of Roc (COR) Domain-Derived Peptides Inhibit Leucine-Rich Repeat Kinase 2 (LRRK2) Dimerization
Source: ACS Chem Neurosci. 2023 May 18;14(11):1971–80. doi: 10.1021/acschemneuro.3c00259 (PMC10251477; doi:10.1021/acschemneuro.3c00259)
Supplement: Supplementary file 1 — cn3c00259_si_001.pdf [file cn3c00259_si_001.pdf]

SUPPORTING INFORMATION

Doubly Constrained Peptides COR-Derived Peptides Inhibit LRRK2 Dimerization

Pragya Pathak[a,#], Krista K. Alexander [b,#], Leah G. Helton [b,#], Michalis Kentros[c], Timothy J. LeClair[b], Xiaojuan Zhang[a], Franz Y. Ho[a], Timothy T. Moore[b], Scotty Hall[b], Giambattista Guaitoli[e], Christian Johannes Gloeckner[e,f], Arjan Kortholt[a,d], Hardy Rideout[c], and Eileen J. Kennedy[b,*]

[a.] Department of Pharmaceutical and Biomedical Sciences, College of Pharmacy, University of Georgia, Athens, GA 30602, United States

[b.] Center for Clinical, Experimental Surgery, and Translational Research, Biomedical Research Foundation of the Academy of Athens, 11527 Athens, Greece

[c.] Department of Cell Biochemistry, University of Groningen, Groningen, Nijenborgh 7, 9747AG Groningen, Netherlands

[d.] YETEM-Innovative Technologies Application and Research Centre Suleyman Demirel University, 32260 Isparta, Turkey

[#] co-first authors

* Corresponding author: Eileen J. Kennedy; email: ekennedy@uga.edu

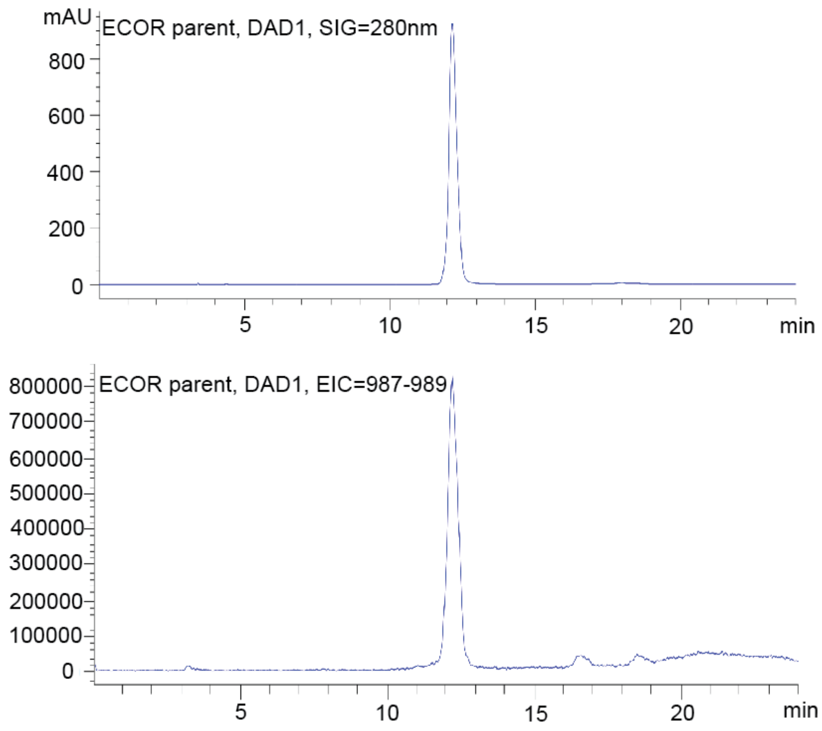

Figure S1. ESI-MS Analysis of FAM labeled ECOR parent. FAM ECOR Parent: (5/6 FAM)-PEG_3- GEGETLLKKWALYSFNDGEEHQKILLDL. Actual mass 3948.8 (Expected mass = 3949.5).

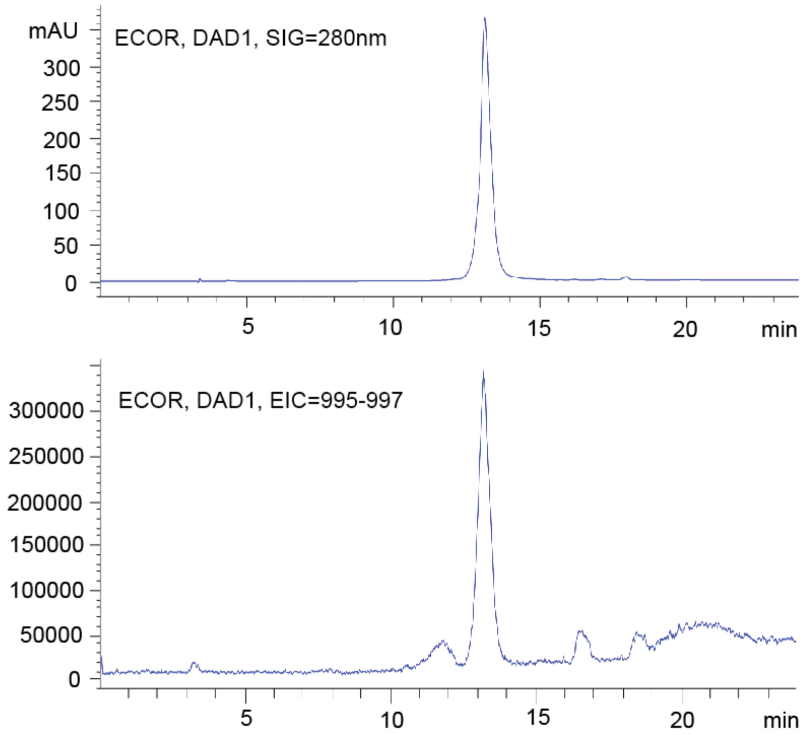

Figure S2. ESI-MS Analysis of FAM labeled ECOR. FAM ECOR: (5/6 FAM)-PEG$_3$-KGEGE*LLK*WALYSFNDGEKH*KKL*KL. Asterisks represent (S)-N-Fmoc-2-(4-pentenyl) alanine. Actual mass 3978.9 (Expected mass = 3979.6).

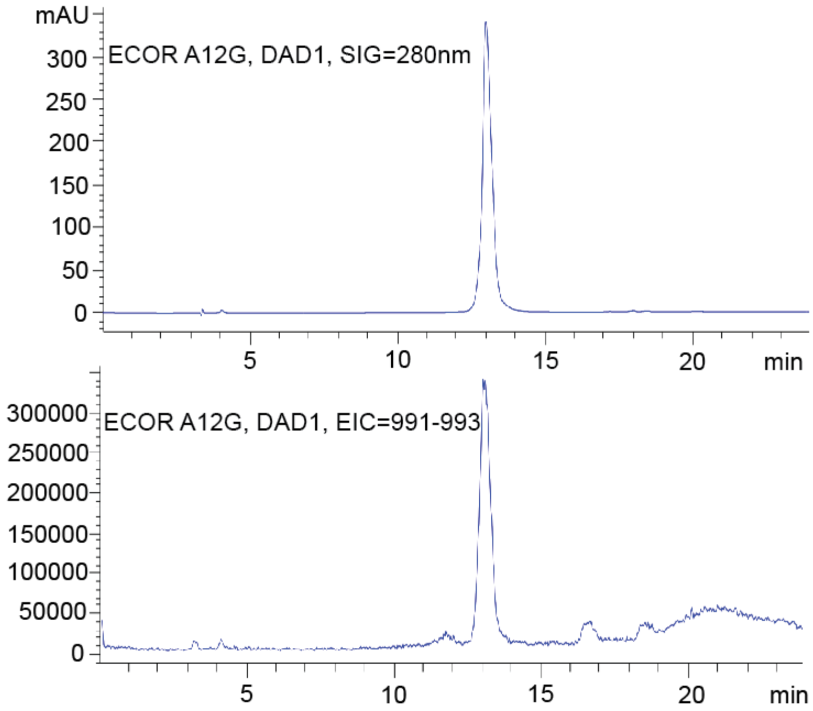

Figure S3. ESI-MS Analysis of FAM labeled ECOR A12G. FAM ECOR A12G: (5/6 FAM)-PEG$_3$- KGEGE*LLK*WGLYSFNDGEKH*KKL*KL. Asterisks represent (S)-N-Fmoc-2-(4-pentenyl) alanine. Actual mass 3965.4 (Expected mass = 3965.6).

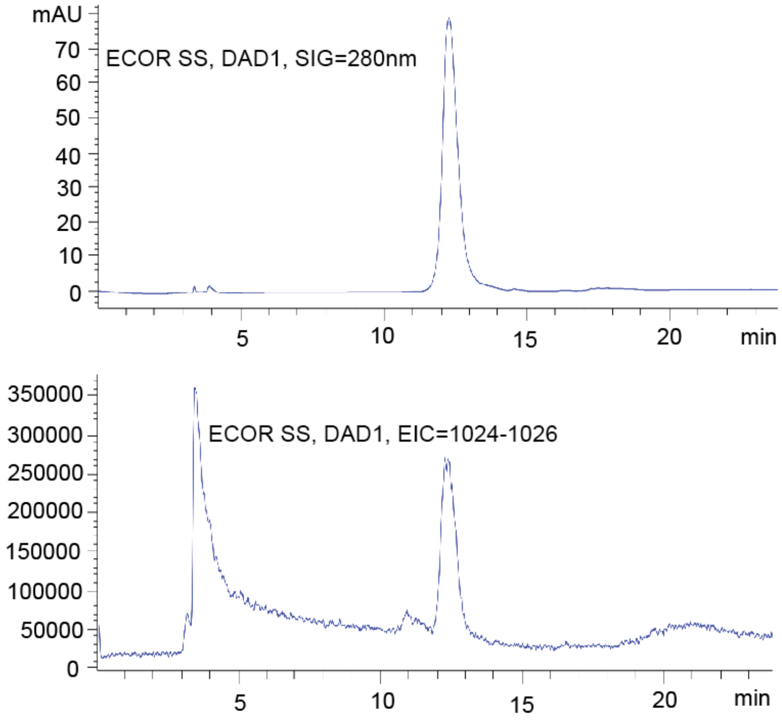

Figure S4. ESI-MS Analysis of FAM labeled ECOR SS. FAM ECOR SS: (5/6 FAM)-PEG$_3$-KGEGE*LLK*WALYSFNDGEKHQKKLLKL. Asterisks represent (S)-N-Fmoc-2-(4-pentenyl) alanine. Actual mass 4099.2 (Expected mass = 4099.7).

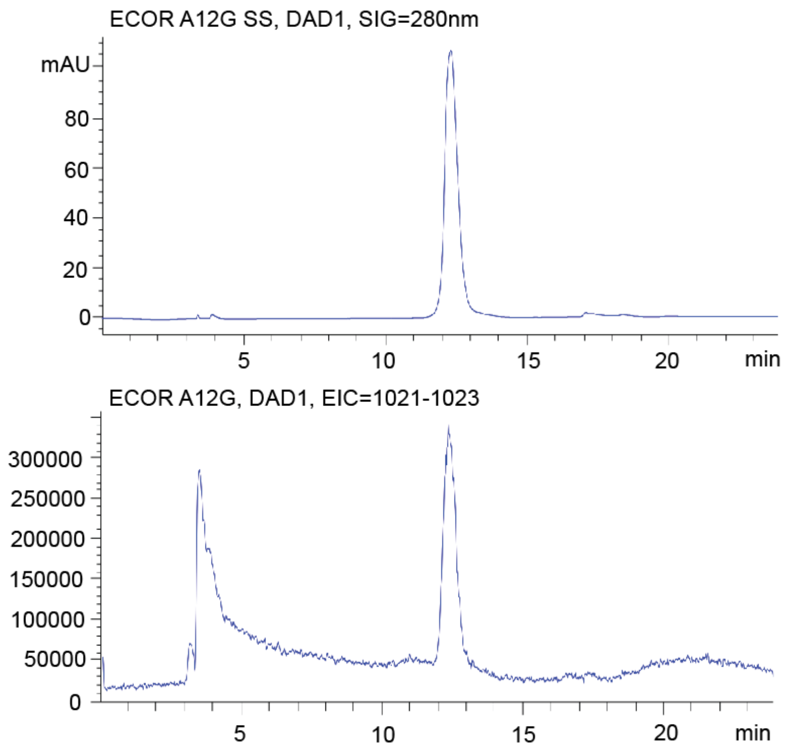

Figure S5. ESI-MS Analysis of FAM labeled ECOR A12G SS. FAM ECOR A12G SS: (5/6 FAM)-PEG$_3$- KGEGE*LLK*WGLYSFNDGEKHQKKLLKL. Asterisks represent (S)-N-Fmoc-2-(4-pentenyl) alanine. Actual mass 4085.2 (Expected mass = 4085.7).

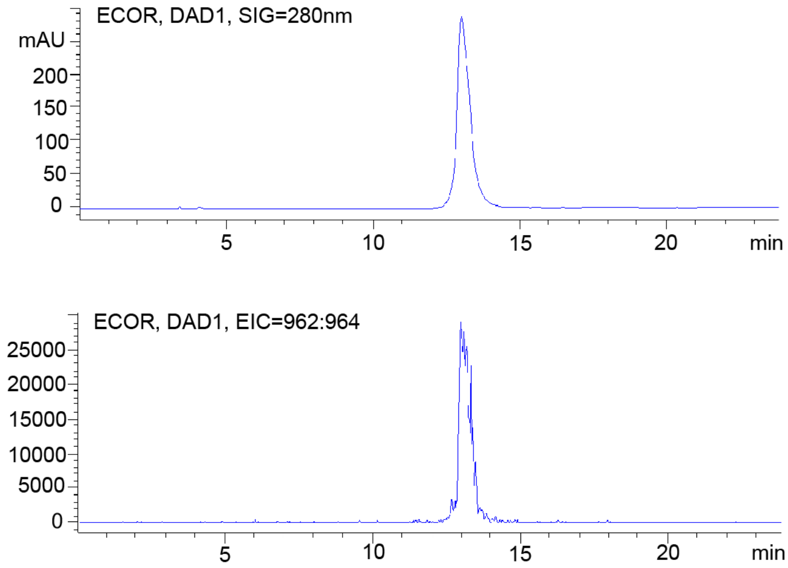

Figure S6. ESI-MS Analysis of Biotin-labeled ECOR. BIO ECOR: (D-Biotin)- PEG_3-KGEGE*LLK*WALYSFNDGEKH*KKL*KL. Asterisks represent (S)-N-Fmoc-2-(4-pentenyl) alanine. Actual mass 3847.1 (Expected mass = 3847.6).

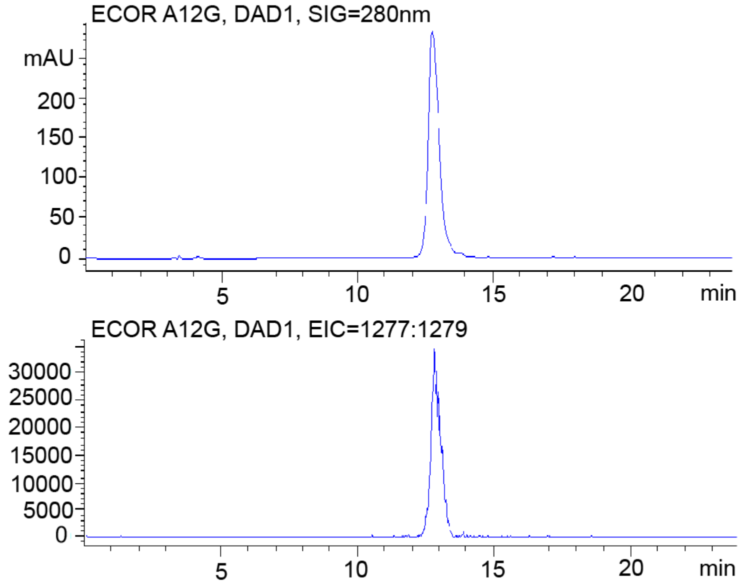

Figure S7. ESI-MS Analysis of Biotin-labeled ECOR A12G. BIO ECOR A12G: (D-Biotin)-PEG$_3$- KGEGE*LLK*WGLYSFNDGEKH*KKL*KL. Asterisks represent (S)-N-Fmoc-2-(4-pentenyl) alanine. Actual mass 3833.0 (Expected mass = 3833.6)

(a)

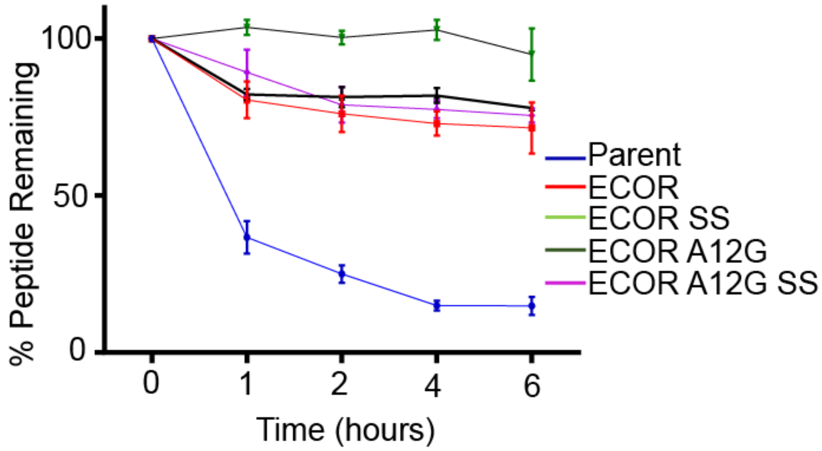

Figure S8. Stapled peptides are resistant to proteolytic degradation in mouse serum. (a) Proteolytic stability was measured for each peptide in mouse serum over a 6-hour time course at 37 °C. The singly and doubly stapled peptides demonstrate considerable proteolytic stability with up to 30% degradation at the 6-hour time point, while the unstapled parent peptide underwent greater than 50% degradation within the first hour. Plots are representative of triplicate experiments.

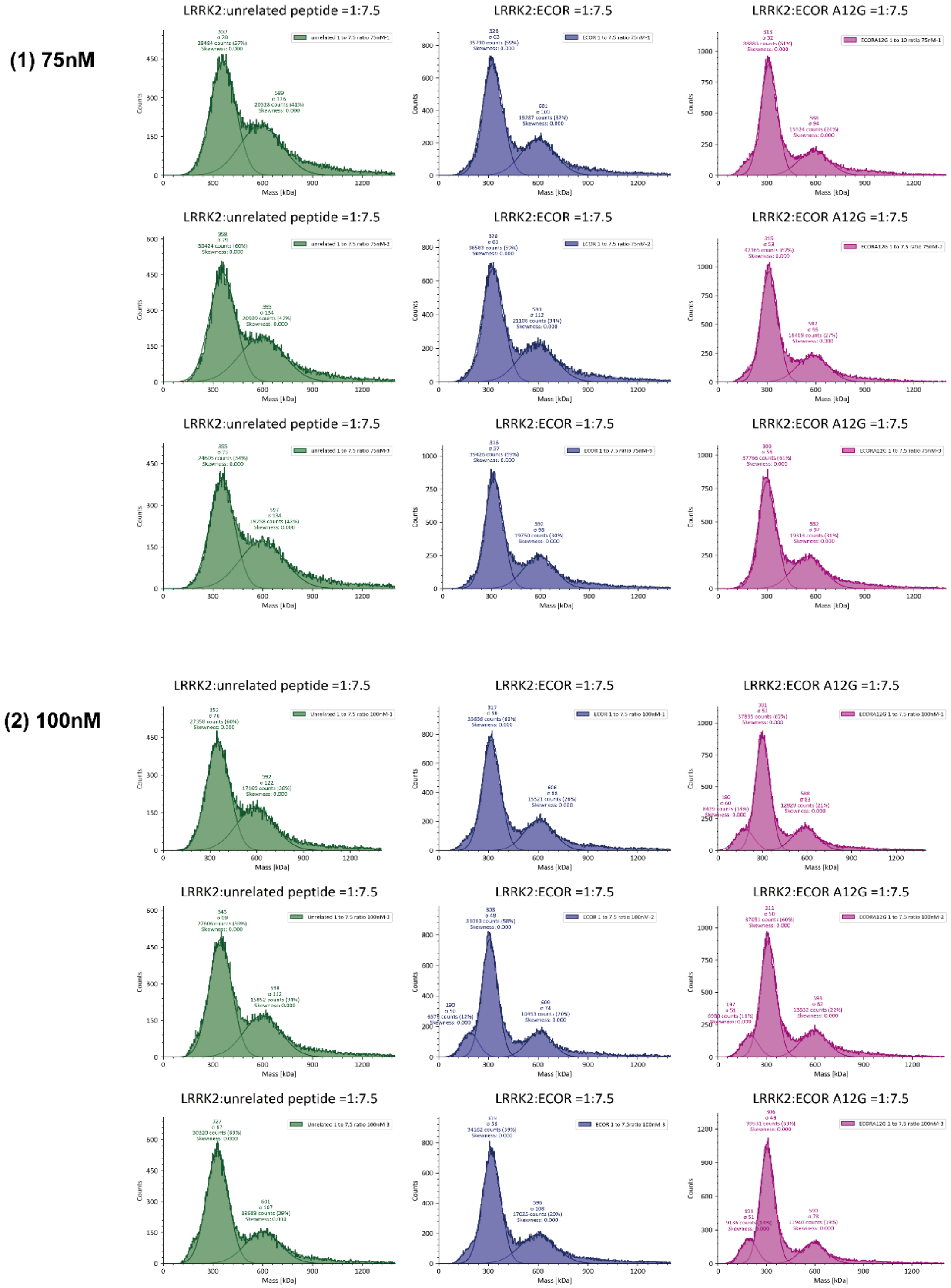

Figure S9. Doubly constrained peptides downregulated LRRK2 dimerization in Mass Photometry. The Mass Photometry diagrams indicate the molecular weight and corresponding particle percentage. Diagrams indicate data for both concentrations of peptide: 75nM and 100nM. Each condition was measured 3 times.
